# Exploring the utility and acceptability of Faecal immunochemical testing (FIT) as a novel intervention for the improvement of colorectal Cancer (CRC) surveillance in individuals with lynch syndrome (FIT for lynch study): a single-arm, prospective, multi-centre, non-randomised study

**DOI:** 10.1186/s12885-022-10217-y

**Published:** 2022-11-07

**Authors:** Anne Lincoln, Sally Benton, Carolyn Piggott, Bernard V. North, Jane Rigney, Caroline Young, Philip Quirke, Peter Sasieni, Kevin J. Monahan

**Affiliations:** 1 Comprehensive Cancer Centre, King’s College London, London, UK; 2 NHS Bowel Cancer Screening South of England Hub, Royal Surrey County Hospital NHS Foundation Trust, Guildford, UK; 3 Pathology & Data Analytics, Leeds Institute of Medical Research at St. James’s, University of Leeds, Leeds, UK; 4 The Lynch Syndrome and Family Cancer Clinic, St Mark’s Hospital and Academic Institute, Harrow, UK; 5 Imperial College London, London, UK

**Keywords:** Lynch syndrome, Colorectal Cancer surveillance, Bowel Cancer surveillance, Mismatch repair deficiency, Faecal immunochemical testing (FIT), Microbiome

## Abstract

**Background:**

Lynch Syndrome (LS) is an inherited cancer predisposition syndrome defined by pathogenic variants in the mismatch repair (MMR) or *EPCAM* genes. In the United Kingdom, people with LS are advised to undergo biennial colonoscopy from as early as 25 until 75 years of age to mitigate a high lifetime colorectal cancer (CRC) risk, though the consideration of additional surveillance intervention(s) through the application of non-invasive diagnostic devices has yet to be longitudinally observed in LS patients. In this study, we will examine the role of annual faecal immunochemical testing (FIT) alongside biennial colonoscopy for CRC surveillance in people with LS.

**Methods/design:**

In this single-arm, prospective, non-randomised study, 400 LS patients will be recruited across 11 National Health Service (NHS) Trusts throughout the United Kingdom. Study inclusion requires a LS diagnosis, between 25 and 73 years old, and a routine surveillance colonoscopy scheduled during the recruitment period. Eligible patients will receive a baseline OC-Sensor™ FIT kit ahead of their colonoscopy, and annually for 3 years thereafter. A pre-paid envelope addressed to the central lab will be included within all patient mailings for the return of FIT kits and relevant study documents. A questionnaire assessing attitudes and perception of FIT will also be included at baseline. All study samples received by the central lab will be assayed on an OC-Sensor™ PLEDIA Analyser. Patients with FIT results of ≥6 μg of Haemoglobin per gram of faeces (f-Hb) at Years 1 and/or 3 will be referred for colonoscopy via an urgent colonoscopy triage pathway.

16S rRNA gene V4 amplicon sequencing will be carried out on residual faecal DNA of eligible archived FIT samples to characterise the faecal microbiome.

**Discussion:**

FIT may have clinical utility alongside colonoscopic surveillance in people with LS. We have designed a longitudinal study to examine the efficacy of FIT as a non-invasive modality. Potential limitations of this method will be assessed, including false negative or false positive FIT results related to specific morphological features of LS neoplasia or the presence of post-resection anastomotic inflammation. The potential for additional colonoscopies in a subset of participants may also impact on colonoscopic resources and patient acceptability.

**Trial registration:**

Trial Registration: ISRCTN, ISRCTN15740250. Registered 13 July 2021.

## Background

Lynch Syndrome (LS) is an inherited cancer predisposition disorder associated with an increased lifetime risk of gastrointestinal, gynaecological, and other cancers. It is characterised by the presence of pathogenic variants in the mismatch repair (MMR) genes, *MLH1, MSH2, MSH6, PMS2*, or a deletion in *EPCAM*, which regulates *MSH2* gene expression [1].

Colorectal cancer (CRC) is the most common of known LS-associated cancers, with cumulative incidence which is gene-specific and shown to steadily increase by age. By age 70 years, pathogenic variants in *MLH1* and *MSH2* convey a 46 and 35% risk of CRC development, respectively, despite colonoscopic surveillance, whereas those with pathogenic variants in *MSH6* or *PMS2* observe markedly lower cumulative incidence rates by the same age: *MSH6* (20%) and *PMS2* (0-1%) [2]. The varied risk observed between LS-associated pathogenic variants within the MMR pathway may be a reflection of both gene-specific risk, and/or gene-specific effectiveness of colonoscopic surveillance, especially in *PMS2* carriers [3].

In considering the respective cumulative lifetime risks of CRC, the effectiveness of routine surveillance is therefore paramount to ongoing clinical care and management in this patient population [4]. In the UK, surveillance via colonoscopy is recommended at least once every 2 years (biennially) for LS patients. For LS patients with pathogenic variants in *MLH1* and/or *MSH2*, biennial colonoscopic surveillance is recommended to begin at age 25, whereas individuals with pathogenic variants in *MSH6* and/or *PMS2* are advised to commence colonoscopic surveillance at 35 years of age. Colonoscopic surveillance stops at 75 years of age for all LS patients, regardless of MMR pathogenic variant [5].

Though a steady increase in survival has been observed in recent years for LS patients with a prior history of CRC [6], the requirement of up to 25 colonoscopies throughout one’s lifetime is both invasive and resource intensive. Moreover, recent literature has challenged current surveillance guidelines following the publication of prospective data which has observed a similar colorectal cancer stage distribution amongst LS patients irrespective of time since their last colonoscopy [2, 7, 8].

### Rationale for current study

Presently, FIT is used as a non-invasive triage tool for referring people for colonoscopic (or other) investigation in asymptomatic patients invited to participate in national bowel cancer screening programmes, as well as in symptomatic patients presenting to primary care with symptoms suggestive of CRC who require urgent colonoscopy [9, 10]. The utility of FIT for the purpose of surveillance in patients with Lynch Syndrome, however, has not previously been formally assessed to our knowledge.

During the COVID-19 pandemic, an emergency national clinical service utilising FIT was implemented by our team for people with LS within England, entitled “*Rapid evaluation of Faecal immunochemical testing (FIT) levels in individuals with a Lynch Syndrome pathogenic variant to determine a revised threshold for colonoscopy in response to the COVID-19 pandemic* (Short Title: “Rapid FIT for Lynch”) [11]. Data from this emergency service has provided important preliminary insights into the design of the research study described in this paper. In this response to the COVID-19 pandemic, eligible LS patients were mailed a FIT kit in lieu of their standard surveillance colonoscopy, which may have been cancelled or indefinitely postponed, as a way of escalating priority of suspected high-risk patients (as defined by having a FIT result ≥10 μg of haemoglobin (Hb) per gram of faeces (f-Hb)).

Overall, an uptake rate of 63.7% was observed (375/588) for those LS patients who had returned their FIT kit to the laboratory for analysis. We also observed earlier diagnoses of CRC in patients whose investigations were escalated after a positive FIT.

Given the acceptable uptake rate of FIT in this LS patient cohort, we believe that a structured programme enabled by longitudinal research with annual FIT alongside biannual colonoscopy is warranted to sufficiently examine the efficacy of FIT as an additive, non-invasive diagnostic modality for the purposes of surveillance in patients with Lynch Syndrome.

### Objectives

#### To evaluate the specificity of FIT in patients with lynch syndrome

The observation of true negative FIT results for participants will be made through the confirmation of no visible advanced adenomas (AA’s) or CRC at time of colonoscopy and subsequent negative (normal) pathology reports for patients with preceding negative FIT results (< 6 μg/g f-Hb).

#### To identify and explore acceptability, views, and preferences of FIT in this patient population

A qualitative assessment of patient baseline questionnaires, which are adherent to a theoretical framework for the acceptability of novel or self-sampling healthcare interventions [12, 13]. This questionnaire will be distributed to patients as part of their baseline (Year 0 (Y0)) mailing.^1^

#### To evaluate the sensitivity of FIT for the detection of CRC in patients with lynch syndrome

The observation of true positive FIT results will be confirmed by the detection of CRC at the time of colonoscopy, and subsequent confirmation via pathology reports for patients with preceding positive FIT results (≥6 μg/g f-Hb).

#### To evaluate the sensitivity of FIT for the detection of advanced adenomas (AA’s) in patients with lynch syndrome

Herein, AA’s are characterised as an adenoma measuring ≥10 mm, containing a villous component, or displaying high grade dysplasia [14].

#### To examine and characterise the faecal microbiome of patients with lynch syndrome

The faecal microbiome of participants who have opted in for additional research will be characterised. 16S rRNA V4 gene sequencing will be performed on residual bacterial DNA from archived FIT samples and results analysed according to colonoscopy outcome.

Future amended versions of this protocol may also allow for the assessment of cancer-associated genetic changes in human DNA from collected stool, which has the potential to improve the accuracy of microbiome-based tests.

#### To observe the incidence of interval CRCs for lynch syndrome patients with negative FIT results

The rate of potential interval CRCs will be observed in a subset of LS patients with preceding negative FIT results up to the end of the passive follow-up period (Baseline + follow-up years 1-3 + 3 years passive follow-up) for a total of 7 years.

Though the risk of interval CRCs following FIT testing has been previously examined in symptomatic patients [15], interval CRC risk following FIT testing in patients with Lynch Syndrome has yet to be explored.

## Methods

### Study design

This is a single-arm, prospective, non-randomised, multi-centre study. Patients will be recruited from eleven [11] NHS Trusts throughout the UK (London North West University Healthcare NHS Trust; The Newcastle Upon Tyne Hospitals NHS Foundation Trust; Guy’s and St Thomas’ NHS Foundation Trust; St George’s University Hospitals NHS Foundation Trust; Manchester University NHS Foundation Trust; Birmingham Women’s and Children’s NHS Foundation Trust; Oxford University Hospitals NHS Foundation Trust; Queen Elizabeth Hospital, University Hospitals Birmingham NHS Foundation Trust; Ninewells Hospital, Dundee, NHS Tayside; University Hospitals Bristol NHS Foundation Trust; and University Hospitals Plymouth NHS Trust). Recruitment for this study commenced in September 2021 with active recruitment anticipated to end on 31st March 2023, or when overall accrual reaches *n =* 400.

With eleven participating study sites in the sole intervention arm, we anticipate approximately 2745 Lynch Syndrome patients. Based on published data from England’s national screening programme FIT pilot study [16] of 3933 “prevalent first-time invitees”, 3686 negative tests were identified in a subgroup of patients whose colonoscopy data was unavailable. In addition, 247 FIT positive tests were identified of which 6 individuals were subsequently diagnosed with CRC following colonoscopy. In order to estimate how many cancers may have been missed by the absence of colonoscopies in those 3686 invitees with negative FIT tests, we note that Li et al. [17] estimates that a threshold of 20 μg haemoglobin (Hb)/g of faeces has 82.2% sensitivity to identify CRC, indicating that an estimate of the total number of cancers in the 3933 individuals would be 6/0.822 = 7.3 cancers. Thus, in addition to the 6 CRC cases with FIT positive results as seen in the FIT pilot study, another 1-2 CRC cases are likely to be undiagnosed in FIT negative (false negative) individuals. Therefore, if we assume a worst case occurrence of 2 false negative cases (i.e., 2 cancers in the 3686 FIT negative tests), then we can estimate that 3933 (total n of “prevalent first-time invitees”)-6-2 = 3925 individuals are without CRC of whom 3686 – 2 = 3684 have FIT negative results, leading to an estimated specificity of 94% (3684 / 3925) in the patient population for this ‘FIT for Lynch Study’.

Assuming approximately three quarters of the eligible 2745 LS patients are due for colonoscopy within the 18 month recruitment period (2745 × 0.75 = 2059), and in considering the projected response/uptake rate of ~ 63%, (which has since been observed in the same patient population as part of the continuing NHS England clinical service evaluation [18]), it is estimated that 1297 individuals will return a FIT sampling kit.

On aiming to recruit 400 Lynch Syndrome patients on surveillance (when considering existing available funding), we anticipate 352 (88%), will not have detectable advanced neoplasia and will be used to evaluate the specificity. Assuming true specificity of 94%, then the width of a 95% confidence interval will be approximately 5%. These numbers would also provide 87% power to show that the specificity is at least 90%. This is based on a one-sided one-sample score test of proportions with a 5% significance level using STATA/SE v. 17.0.

We will employ a modified intention-to-treat (mITT) approach whereby participants who return at least 1 FIT kit and have an outcome from colonoscopy are available for analysis. Participants whose colonoscopy result is unavailable are likely to be relatively uncommon when considering the high rate of colonoscopy surveillance adherence in this patient population [19, 20]. Nonetheless, we will report the frequency of those without colonoscopy and the frequencies of those returning 0, 1 or 2 FIT kits.

### Study population: inclusion and exclusion criteria

Inclusion criteria: Patients will have a confirmed pathogenic variant in any four MMR genes (*MLH1, MSH2, MSH6, PMS2),* or have a confirmed pathogenic variant in *EPCAM.* For those with pathogenic variants in *MLH1* or *MSH2,* the age requirements for eligibility will be between 25 and 73 years, and for patients with pathogenic variants in *PMS2, MSH2* or *EPCAM* must be between the ages of 35 – 73 years of age. Eligible patients must also have an upcoming (within the year of active recruitment) standard of care (SOC) colonoscopy appointment at any NHS Trust. Age criteria is defined by gene-specific colonoscopy eligibility, with the upper maximum age limit in this instance defined by the upper age limit for LS surveillance guidelines (75 years of age) [5], with 2 years subtracted to accommodate a surveillance SOC colonoscopy at Study Year 2.

Exclusion criteria: Individuals who have previously undergone a subtotal or total colectomy and/or those who are unable to provide consent in English will be excluded from this study.

### Baseline recruitment

Potential eligible participants will be identified by the local PI and study team of the respective participating NHS Trusts via a i) Lynch Syndrome registry ii) local regional genetics service databases iii) through linked endoscopy clinical records held within their local Regional NHS Trusts and/or iv) through the completion of a reply slip that may be sent from a participating Regional Genetics Service (study site) direct to their LS patients, which shall be sent along with a patient inquiry letter as part of an initial optional patient mailing, as detailed below. Inclusion and exclusion criteria will be verified by the hospital clinical records. Ethically-approved advertisement flyers and patient informational posters will be shared amongst Lynch Syndrome support groups and related networks as an addition strategy to inform eligible patients about the study.

The Research laboratory at the Bowel Cancer Screening South of England Hub (referred to as the “Southern Hub laboratory”), based at the Royal Surrey County NHS Foundation Trust in Guildford, will act as the central laboratory for this study. Participating study sites will email an excel spreadsheet containing patient details by secure nhs.net email patients meeting full inclusion criteria for the study. Southern Hub laboratory research staff will compile baseline mailings (Year 0) for each eligible patient which will be mailed direct to eligible patients within 7-30 days prior to their next scheduled standard of care (SOC) colonoscopy. This specific time frame (7-30 days) was devised by considering FIT kits to be received by eligible patients prior to commencing bowel preparations for colonoscopy, and in considering potential mail delays or delivery disruptions.

Prior to the distribution of a baseline mailing, Southern Hub laboratory research staff will assign a Study ID to each eligible patient which will be recorded within a secure master database internal to the Southern Hub research laboratory and restricted solely to assigned study staff.

The baseline mailings will comprise a i) study invitation letter ii) a faecal immunochemical test (FIT) kit (OC-Sensor™, Eiken Chemical Co. Ltd., Tokyo, Japan) enclosed within a biospecimen sample bag iii) a consent form, which will include an opt-in to allow for additional research as part of the exploratory aim to examine the faecal microbiome of LS patients, iv) pictorial and written instructions for collection of the faecal sample (FIT instruction sheet), v) a participant questionnaire, vi) a participant information sheet (PIS), and vii) a return envelope with prepaid postage for return to the Southern Hub.

For any participating Regional Genetics Services (study sites) who are unable to access requisite colonoscopy appointment details for their LS patients, an optional patient inquiry letter and accompanying reply slip may be mailed directly from said study sites to their LS patients who have met all preceding eligibility criteria.

Consent to this study will be implicit upon return of a completed FIT kit at Baseline (Year 0), though all participants are free to withdraw at any time. Any study data and/or relevant (pseudo-anonymised) clinical data that has been collected until this point, however, will remain as part of the study database and therefore retained by the study team.

Upon receipt of any study FIT kits (Baseline + Years 1-3), a numerical sample ID, which will be consistent with the accompanying barcode found on each FIT kit label, will be assigned and linked to the participant’s previously assigned study ID. Each returned FIT kit will be analysed on one of two OC-Sensor™ Pledia analysers within 14 days of sample collection.

F-Hb results will be reported down to the limit of detection (LoD) (1 μg/g f-Hb), as recommended by Fraser and Benton [15, 21, 22].

Pseudo-anonymised data, including Study ID’s, relevant demographic variables, and f-Hb concentration results of study participants, will be recorded by assigned Southern Hub laboratory research staff within respective electronic clinical report forms (eCRF’s) as part of the FIT for Lynch Study database. This study database will use REDCap electronic data capture tools hosted by the coordinating centre, King’s College London (KCL) [23, 24].

### Post-baseline study years 1-3: contact & mailings

**Fig. 1:**
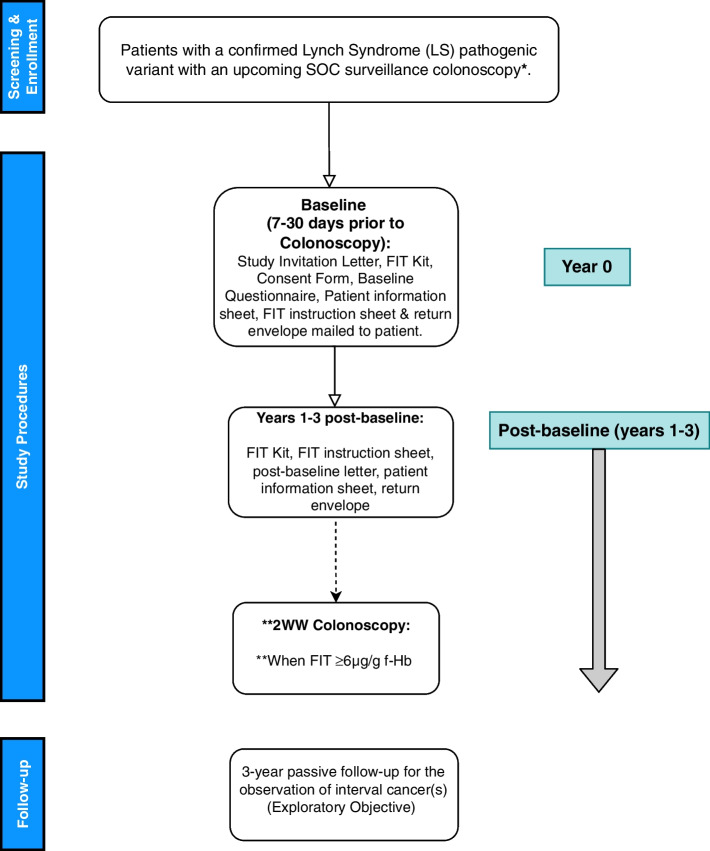
FIT for Lynch Study Schematic. * = Per the inclusion criteria, eligible patients with *MLH1* OR *MSH2* pathogenic variants must be between 25 and 73 years of age, whereas eligible patients with pathogenic variants in *PMS2, MSH6,* or *EPCAM* must be between 35 and 73 years of age

Following active recruitment and baseline period (Year 0), participants will be re-contacted by Southern Hub laboratory research staff via mail on an annual basis for an additional 3 years (Study Years 1-3) after baseline (Year 0). Subsequent mailings for enrolled participants for Years 1-3 will be comprised of the following items and materials: i) a participant post-baseline letter, ii) a participant information sheet, iii) an OC-Sensor™ FIT kit enclosed within a biospecimen bag, iv) a FIT instruction sheet, and a v) return envelope with prepaid postage. Mailing of study materials and FIT kits for study participants at Study Year 2 will be sent ahead of their SOC colonoscopy at this time point (in line with the UK’s LS surveillance guidelines) also within a 7–30-day window (See Fig. 1 and Table 1) .

**Table 1:** Study schedule of events (baseline & years 1-3)

Study Participants (SP)	Active Recruitment (initial 12 months post study start)	Screening (7-30 days prior to colonoscopy)	Baseline (7-30 days prior to colonoscopy)	1-year post-baseline	2-years post-baseline	3-years post-baseline
Eligibility assessment		x				
^α^ Patient Inquiry Letter and reply slip	x					
FIT Kit, FIT instruction sheet, patient letter, patient information sheet (mailed)			x	x	x	x
Study invitation letter, consent form, baseline questionnaire, patient information sheet			x			
Post-baseline letter				x	x	x
^‡^Follow-up Questionnaire						x
SOC colonoscopy			+		+	
**Colonoscopy referred via 2WW				**+		**+

x Research related procedure.

+ SOC procedure (as per recommended guidelines)

α Optional mailing for study sites who may not have access to requisite colonoscopy appointment data (direct mailing between said study sites and LS patients).

^**‡**^ A follow-up questionnaire to assess evolving perceptions and attitudes towards FIT is presently under development as of the time of this writing and will be forthcoming for inclusion within the study design ahead of its planned distribution to study participants at Year 3

** Enrolled patients shall be referred for a colonoscopy (by their consultant) via the two-week wait (2WW) pathway performed at intervening years between SOC biennial colonoscopy (Years 1 &3) *only* in the event their FIT result ≥6 μg/g f-Hb

Returned FIT kits in post-baseline Study Years 1-3 will follow the same standard operating procedure for analysis and eCRF reporting as detailed above for baseline mailings and study samples.

### FIT kit results Reporting & Urgent Colonoscopy Triage for eligible study participants (SP) at study years 1 & 3

F-Hb results to come from this study will not be directly disclosed to participants. Site Principal Investigator’s (PI’s) will receive electronic reports of f-Hb results if ≥6 μg/g study participants’ directly from the Southern Hub laboratory via NHS secure and encrypted emails in Years 1 and 3 (the intervening years between a study participant’s SOC colonoscopy) to inform triage for colonoscopy. FIT positivity in study participants will be determined by the limit of quantification (LoQ) at the pre-determined threshold of ≥6 μg/g f-Hb [22].

Although it is anticipated that only a small minority of study participants may meet criteria for an additional interval colonoscopy at Years 1 & 3 (approximately 10%, based on early data as part of a preceding emergency clinical service evaluation), those that are identified as “FIT positive” at the specified study periods will be contacted by their respective local PI to discuss their FIT results and advised to proceed with a colonoscopy which will be triaged via the NHS’ urgent two-week-wait (2WW) referral pathway.

Throughout the duration of the study, endoscopy and pathology reports will be securely sent from respective study sites to the coordinating centre for the abstraction of relevant outcome data which will be stored and managed within the FIT for Lynch REDCap database.

### Compliance

To ensure a high adherence to the trial protocol and the completion of annual FIT kits, eligible patients receive clear FIT kit instructions, as well as detailed study materials and the FIT kit on an annual basis throughout the course of the study (Fig. 1). For post-baseline periods (Years 1-3), participants receive a personalised post-baseline letter which serves as a reminder of their study participation and to request the timely return of their FIT kit at the specified study point. For baseline (Year 0) and Year 2, participants are encouraged to return their FIT kit to the Southern Hub ahead of their scheduled SOC colonoscopy and prior to beginning their bowel prep by utilising an enclosed pre-paid envelope which allows for simplicity, ease, and a cost-free method in delivering study samples.

### Data collection and planned analysis

Colonoscopy and/or histopathology reports will be requested for all participating individuals who return a FIT kit throughout the duration of the trial (Baseline + Years 1-3) as part of the outcome data required for analysis. After the trial has ended, and dependent on the procurement of additional research funds, we will continue to passively observe a subset of trial participants who had preceding negative FIT results to discern potential incidence of interval advanced colorectal neoplasia (ACN) over the course of 3 years. The study has received written approval from the Research Ethics Committee (REC) of Clinical Research of each participating region (REC Reference: 21/YH/0066).

A secure and encrypted database will be constructed with a unique study ID for each participant and routinely checked for errors. A summary of study variables to be collected are reflected within Table 2.

**Table 2:** Study measurements

Participant	
Study ID, DOB^a^, sex, ethnicity, NHS Lynch Syndrome genotype, local PI and participating NHS Trust.	
FIT Data	
Dates of participant mailings sent from the Southern Hub (baseline and years 1-3), FIT results (f-Hb for baseline and years 1-3.	
Clinical^a^	
Date of colonoscopy at baseline and years 1-3 + follow-up years 4-6 for the subset of eligible patients, histological features and staging (where applicable) of biopsied specimen(s) at time of colonoscopy, anastomosis (Y/N), gastrointestinal (GI) comorbidities (Y/N), History of CRC (Y/N), date of CRC (where applicable), prior GI surgery (Y/N), GI surgery type (where applicable), other cancer(s) (Y/N), bowel prep quality, polyp retrieval (Y/N), colonoscopy key performance indicators (KPI) met (Y/N), aspirin use (Y/N), aspirin dosage (where applicable), additional medications (Y/N), additional medication details and dosage (where applicable).	

^a^Data to be collected only for those who return a sample i.e., consent

Both interim and final datasets will be created and analysed within Stata statistical software. The REDCap database and related .csv files will be imported into Stata for final cleaning and analysis.

The interim dataset will be developed and analysed within Stata by the Project Manager and PhD student, Anne Lincoln, as part of her PhD thesis. The preliminary data will be comprised of all available baseline data (e.g., FIT results, baseline questionnaire responses, and endoscopy and histopathology results (where applicable) for SOC colonoscopies at baseline)).

Additional data will be collected from participant questionnaires included in the baseline mailing to address the secondary study objective.

### Outcomes and endpoints

A detailed description of the objectives and the measurement of study outcomes is provided herein (see Table 3).

**Table 3:** Measurement timepoints of study objectives

*Study Objectives*		*Measurement Timepoints*				
*Objective Type*	*Objective Summary*	*Baseline (Y0)*	*Y1*	*Y2*	*Y3*	*Passive Follow-Up (Y4-Y7)*
Objective 1 (Primary)	To evaluate the specificity of FIT for the detection of CRC and AA’s in patients with LS.	x	x	x	x	
Objective 2 (Secondary a)	To identify and explore acceptability, views, and preferences of FIT in this patient population	x				
Objective 3 (Secondary b)	To evaluate the sensitivity of FIT for the detection of CRC in patients with LS	x	x	x	x	
Objective 4 (Secondary c)	To evaluate the sensitivity of FIT for the detection of AA’s in patients with LS	x	x	x	x	
Objective 5 (Exploratory)	To examine and characterise the faecal microbiome of patients with LS.	x	x	x	x	
Objective 6 (Exploratory)	To observe the incidence of interval CRCs for LS patients with FIT negative results (< 6 μg/g f-Hb)					x^a^

^a^*This outcome will be measured in a subset of study participants*

### Primary outcomes

#### Objective 1 (primary)

Primary outcomes are evaluated at all time points (Baseline (Y0) + Years 1-3 (Y1-Y3)) and will entail the assessment of true negative and false negative rates in this participant cohort to observe ≥90% specificity. In this context, true negative is defined as having no visible CRC or AA’s at time of colonoscopy and a subsequent normal or “negative” endoscopy and/or pathology report for participants with preceding FIT negative (< 6 μg/g f-Hb) results.

### Secondary outcomes

#### Objective 2 (secondary objective (a))

Secondary outcomes for secondary objective (a) are evaluated at Baseline (Y0) through the qualitative assessment of patient questionnaires which are adherent to a theoretical framework for the acceptability of novel or self-sampling healthcare interventions [12, 13].

#### Objective 3 (secondary objective (b))

Secondary outcomes for secondary objective (b) are evaluated at all time points (Y0 – Y3) and will entail the assessment of true positive and false negative rates in this participant cohort to observe > 87% sensitivity. True positive (TP) cases are defined as the confirmation in the detection of colorectal neoplasia at time of colonoscopy for patients with preceding positive FIT results (≥6 μg/g f-Hb) and will be evaluated at thresholds of 6, 10 and 100 μg/g for patients meeting this criteria of TP.

#### Objective 4 (secondary objective (c))

Secondary outcomes for secondary objective (c) are evaluated at all time points (Y0 – Y3) and will entail the assessment of true positive and false negative rates in this participant cohort to observe > 87% sensitivity. True positive cases in this context are defined as the confirmation in the detection of AA’s alone at time of colonoscopy for patients with preceding positive FIT results (≥6 μg/g f-Hb) and will be evaluated at thresholds of 6, 10 and 100 μg/g for patients meeting this criteria of TP.

### Exploratory outcomes

#### Objective 5 (exploratory)

16S ribosomal RNA (rRNA) gene V4 amplicon sequencing will be carried out on residual faecal DNA of eligible archived FIT study samples to characterise the faecal microbiome.

#### Objective 6 (exploratory)

The assessment of interval CRC’s will be examined after study end and during the passive follow-up period (Y4-Y7) in a subset of LS patients who had FIT negative results (< 6 μg/g f-Hb) throughout the course of the study (baseline + Y1-Y3).

### Monitoring

A mixture of on-site visits and central monitoring will be utilised throughout the life cycle of this trial. On-site visits will be conducted by the trial coordinator of the coordinating site, which will allow for the data to be verified against source documents, ensure essential documents are present in the Investigative Site File (ISF), and FIT kits have been delivered, stored, and dispensed appropriately. Centralised (remote) monitoring will be conducted by a named delegate of the Chief Investigator’s (CI’s) team and will allow the CI and Study Sponsor to maintain oversight of the trial. The central monitoring delegate will be listed on the trial delegation log, trained in Good Clinical Practice (GCP) and monitoring procedures specific to the coordinating site. Trial Management Group meetings will be held on a monthly basis to provide overall guidance and trial oversight.

Since the trial intervention, OC-Sensor™, is well characterised, non-invasive and of low risk, and tested as a supplemental modality to existing CRC surveillance practices for patients with LS, a Data Monitoring Committee has not been set up.

We do not anticipate any major safety concerns for the study, as faecal self-sampling using FIT kits has been safely performed in several other studies to date. Adverse events are therefore anticipated to be exceedingly minimal as the Clinical Laboratory Improvement Amendments (CLIA) waived FIT kit, OC-Sensor™, which will be utilised for the purposes of this research, is non-invasive and may be completed by participants in the comfort of their own home. Relatedly, serious adverse events (SAEs) are not anticipated and will therefore not be reported.

### Quality control and assurance

Internal standard operating procedures will be strictly adhered to for the development, implementation, documentation, and analyses of the clinical trial. Moreover, all relevant regulations will be observed. Any proposed changes to the study conduct, design, or management will be notified to the original approving Research Ethics Committee (REC), and any other applicable regulatory authority via a substantial amendment. Changes to the study will not be implemented until a REC and any other relevant authority (e.g., Health Research Authority (HRA) amendment approval) has been obtained. Following REC approval, any approved study changes will be immediately communicated to the participating study sites and their respective Research & Development delegates for local approval and acknowledgement.

The study will comply with the General Data Protection Regulation (GDPR) which requires data to be anonymised / pseudo anonymised as soon as it is practical to do so. Identifiable fields will be removed and replaced with the participant’s unique Study ID. Identifiable data will not be included in the files provided for analysis.

The findings from this study will be made publicly available through publication or other dissemination tools without any unnecessary delay, and an honest and accurate transparent account of the study will be provided at that time.

## Discussion

As the implementation of our emergency national clinical service evaluation has elucidated thus far, the utility of FIT may present clinical value for patients with Lynch Syndrome as a means of non-invasive CRC surveillance in intervening years between colonoscopy, and as an efficient mechanism in risk-stratifying the highest risk patients within this patient population [18]. Though qualitative analysis is presently underway in understanding LS patient attitudes and perceptions towards this diagnostic device, the patient response rate (63%) exemplified a favourable response overall. Provided the short duration of this clinical service as an emergency measure to the COVID-19 pandemic, further investigation on the efficacy of this novel intervention is warranted and may be enabled by this longitudinal, multi-centre, prospective research study.

In addition to elucidating overall efficacy of FIT in patients with Lynch Syndrome, we aim to characterise the faecal microbiome of individuals with Lynch Syndrome (as outlined in Objective 4) through residual faecal DNA. The data to be collected from this study may facilitate deeper insights into the unique bacterial communities which reside in the microbiome of LS patients which may have important clinical implications. Moreover, we envisage subsequent versions of this protocol may allow for the assessment of cancer-associated genetic changes in human DNA from stool which have the potential to improve the accuracy of a microbiome-based test.

At the time of this writing, eleven NHS Trusts throughout England and Scotland have been included as study sites and active recruitment is underway. Nonetheless, we recognise that this study may present certain limitations or issues, such as the potential for additional colonoscopies during intervening years between biennial surveillance colonoscopies, which may be considered invasive and potentially cause harm to no benefit to the patient.

Considering the unique histopathology and morphology of LS-associated neoplasia (with a higher frequency of flat/subtle neoplasia) [25], the sensitivity of FIT may be lower in comparison to the FIT sensitivity rates depicted within population-based screening programmes. The rate of false positives findings may also be slightly elevated when considering for example the prevalence of anastomotic inflammation from previous CRC resection in this patient population.

Moreover, patients may perceive a negative (< 6 μg/g) FIT result as a conclusive finding which may potentially impact subsequent adherence to biennial colonoscopy for subsequent year(s). For this reason, FIT results will not be passed directly to the patient, though there is a possibility that participants may discern a negative FIT finding with the lack of a colonoscopy referral via the two-week wait (2ww) pathway in Years 1 & 3. It is also to be noted that any 2ww referred colonoscopies for eligible individuals in Years 1 & 3 may be conducted by a non-family cancer endoscopist, or someone unfamiliar with Lynch Syndrome and/or the research study, which may ultimately result in inconsistent or suboptimal colonoscopies. Despite the potential limitations in which FIT may pose throughout the duration of this study, colonoscopy alone may present constraints as well, such as the development of interval cancer(s) which may only be detectable at a later stage.

In considering the above, in addition to the known age, gene and gender-specific CRC risks that are observed within the LS population, we envisage FIT as an augmentative rather than a replacement modality with the potential to identify interval neoplasia and/or CRCs and it should therefore still be considered and further examined in this high-risk patient population. For these reasons, and in considering the breadth of this multi-Institutional research study, a concerted effort in standardising data collection is of utmost importance and has been prioritised by the research team.

## Data Availability

The dataset used and analysed during the current study will be made available from the corresponding author on reasonable request.

## Abbreviations

FIT: Faecal immunochemical test
CRC: Colorectal Cancer
CN: Colorectal Neoplasia
ACN: Advanced Colorectal Neoplasia
LS: Lynch Syndrome
SOC: Standard of Care
MMR: Mismatch Repair
EPCAM: Epithelial cell adhesion molecule
NHS: National Health Service
REC: Research Ethics Committee
HRA: Health Research Authority
SP: Study Participants
LoD: Limit of Detection
f-Hb: Faecal Haemoglobin
rRNA: Ribosomal Ribonucleic Acid
PI: Principal Investigator
CI: Chief Investigator
ISF: Investigative Site File
GCP: Good Clinical Practice
GDPR: General Data Protection Regulation
mITT: Modified Intention-To-Treat
CLIA: Clinical Laboratory Improvement Amendments
SAE: Serious Adverse Event

## Footnotes

1 A follow-up questionnaire to assess evolving perceptions and attitudes towards FIT is under development as of the time of this writing and will be forthcoming for inclusion within the study design ahead of its planned distribution to study participants at Year 3 (see Table 1).

## References

[1] Genetics Home Reference (2020). Lynch syndrome.

[2] Møller P, Seppälä T, Bernstein I, Holinski-Feder E, Sala P, Evans DG (2017). Cancer incidence and survival in lynch syndrome patients receiving colonoscopic and gynaecological surveillance: first report from the prospective lynch syndrome database. Gut..

[3] Møller P (2020). The prospective lynch syndrome database reports enable evidence-based personal precision health care. Hered Cancer Clin Pract..

[4] Dove-Edwin I, de Jong AE, Adams J, Mesher D, Lipton L, Sasieni P (2006). Prospective results of surveillance colonoscopy in dominant familial colorectal cancer with and without lynch syndrome. Gastroenterology..

[5] Monahan KJ, Bradshaw N, Dolwani S, Desouza B, Dunlop MG, East JE (2020). Guidelines for the management of hereditary colorectal cancer from the British Society of Gastroenterology (BSG)/Association of Coloproctology of Great Britain and Ireland (ACPGBI)/United Kingdom Cancer genetics group (UKCGG). Gut..

[6] Møller P, Seppälä T, Bernstein I, Holinski-Feder E, Sala P, Evans DG (2017). Incidence of and survival after subsequent cancers in carriers of pathogenic MMR variants with previous cancer: a report from the prospective lynch syndrome database. Gut..

[7] Seppälä TT, Ahadova A, Dominguez-Valentin M, Macrae F, Evans DG, Therkildsen C (2019). Lack of association between screening interval and cancer stage in lynch syndrome may be accounted for by over-diagnosis; a prospective lynch syndrome database report. Hered Cancer Clin Pract.

[8] Engel C, Vasen HF, Seppälä T, Aretz S, Bigirwamungu-Bargeman M, de Boer SY (2018). No difference in colorectal Cancer incidence or stage at detection by colonoscopy among 3 countries with different lynch syndrome surveillance policies. Gastroenterology..

[9] Chapman C, Bunce J, Oliver S, Ng O, Tangri A, Rogers R (2019). Service evaluation of faecal immunochemical testing and anaemia for risk stratification in the 2-week-wait pathway for colorectal cancer. BJS Open.

[10] Ng O, Humes D, Rogers R, Tangri A, Oliver S, Chapman C (2017). PWE-027 An interim analysis of the ‘getting fit’ project in Nottingham: integrating faecal immunochemical testing in a two week wait pathway. Gut..

[11] Monahan KJ, Lincoln A, East JE, Benton S, Burn J, DeSouza B, et al. Management strategies for the colonoscopic surveillance of people with lynch syndrome during the COVID-19 pandemic. Gut. 2020. 10.1136/gutjnl-2020-321993 32571974

[12] Sekhon M, Cartwright M, Francis JJ (2017). Acceptability of healthcare interventions: an overview of reviews and development of a theoretical framework. BMC Health Serv Res.

[13] Waller J, McCaffery K, Forrest S, Szarewski A, Cadman L, Austin J (2006). Acceptability of unsupervised HPV self-sampling using written instructions. J Med Screen.

[14] Kim DH, Pickhardt PJ, Taylor AJ (2007). Characteristics of advanced adenomas detected at CT colonographic screening: implications for appropriate polyp size thresholds for polypectomy versus surveillance. AJR Am J Roentgenol.

[15] Pin-Vieito N, Iglesias MJ, Remedios D, Rodríguez-Alonso L, Rodriguez-Moranta F, Álvarez-Sánchez V (2020). Risk of gastrointestinal cancer in a symptomatic cohort after a complete colonoscopy: role of faecal immunochemical test. World J Gastroenterol.

[16] Moss S, Mathews C, Day TJ, Smith S, Seaman HE, Snowball J (2017). Increased uptake and improved outcomes of bowel cancer screening with a faecal immunochemical test: results from a pilot study within the national screening programme in England. Gut..

[17] Li SJ, Sharples LD, Benton SC, Blyuss O, Mathews C, Sasieni P (2021). Faecal immunochemical testing in bowel cancer screening: estimating outcomes for different diagnostic policies. J Med Screen.

[18] Lincoln A, Lincoln A, Benton S, Sasieni P (2021). PTH-27 Risk-stratified FIT for urgent colonoscopy in lynch syndrome: a clinical service throughout the COVID-19 pandemic. Gut..

[19] Newton K, Green K, Lalloo F, Evans DG, Hill J (2015). Colonoscopy screening compliance and outcomes in patients with lynch syndrome. Color Dis.

[20] Bleiker EM, Menko FH, Taal BG, Kluijt I, Wever LD, Gerritsma MA (2005). Screening behavior of individuals at high risk for colorectal cancer. Gastroenterology..

[21] D'Souza N, Georgiou Delisle T, Chen M, Benton S, Abulafi M (2021). Faecal immunochemical test is superior to symptoms in predicting pathology in patients with suspected colorectal cancer symptoms referred on a 2WW pathway: a diagnostic accuracy study. Gut..

[22] Fraser CG, Benton SC (2019). Detection capability of quantitative faecal immunochemical tests for haemoglobin (FIT) and reporting of low faecal haemoglobin concentrations. Clin Chem Lab Med.

[23] Harris PA, Taylor R, Thielke R, Payne J, Gonzalez N, Conde JG (2009). Research electronic data capture (REDCap)--a metadata-driven methodology and workflow process for providing translational research informatics support. J Biomed Inform.

[24] Harris PA, Taylor R, Minor BL, Elliott V, Fernandez M, O'Neal L (2019). The REDCap consortium: building an international community of software platform partners. J Biomed Inform.

[25] Ahadova A, Seppälä TT, Engel C, Gallon R, Burn J, Holinski-Feder E, et al. The "unnatural" history of colorectal cancer in lynch syndrome: lessons from colonoscopy surveillance. Int J Cancer. 2020. 10.1002/ijc.33224 32683684

